# Electroconvulsive therapy: the perspective of the informal caregiver in the decision-making process

**DOI:** 10.1192/j.eurpsy.2024.512

**Published:** 2024-08-27

**Authors:** P.-J. Geerts, S. Abihi, S. Verhaeghe

**Affiliations:** ^1^az groeninge, kortrijk; ^2^ugent, Ghent, Belgium

## Abstract

**Introduction:**

Despite the importance of electroconvulsive therapy (ECT) as treatment, it remains one of the most controversial and misunderstood treatments. Negative media representations, primitive practice in the past and fear for electricity results in fear that extends beyond other therapies. Research on the perspective and role of informal caregivers (IC) in the process of ECT is limited. Most research focuses on relatives’ attitude or knowledge of ECT measured with questionnaires. However, profound understanding of their perspective can facilitate the role of physicians (or psychiatrists) in guiding patients and their IC through the decision-making process of ECT.

**Objectives:**

The aim of this study was to describe the perspective of informal caregivers in the decision-making process in ECT treatment.

**Methods:**

A qualitative phenomenological study was set up. Semi-structured interviews were held with IC of patients who are treated with ECT. **Purposive sampling was based on maximum variation. All interviews were fully transcribed and thematic analyses took place.** Trustworthiness was guaranteed by e.g. researcher triangulation

**Results:**

In nine interviews were held with partners, children and parents of patients. The interviews had a mean duration of 102 minutes and interviewing proceeded until saturation of the most important themes was reached. During the interviews it became clear that the decision-making process of ECT is strongly influenced by the illness-trajectory and context of living with the mental health problems of the patient. IC describe their life and that of the patient as ‘trying to survive’. The proposal of ECT is seen as a way out of this unendurable situation. The perceived responsibility of the IC in the informed consent process to ECT adds to this burden. The IC worry, feel uncertain and fear to do wrong. Nonetheless ECT seems to be a beacon of hope. Trust in the psychiatrist as a competent professional who wants the best for the patient seems more important than having an answer to all of their questions. After the ECT has been started, IC establish a framework to evaluate the side-effects and effectiveness of ECT. This framework is based on how they experience the patient in daily life and on what they define as ‘the patient becoming a bit more himself again’. IC weigh the effects and side-effects to support the continuation of ECT. However, if patients clearly express that they experience side-effects that are too hindering, IC follow the patient if he or she wants to stop ECT.

**Conclusions:**

Our study gives an insight in the perspective of the IC of patients undergoing ECT. It could be helpful for IC if the psychiatric team repeats information stepwise and takes the burden of responsibility perceived by the IC into account. The framework used by IC to evaluate the effects of ECT could be a valuable addition to the clinical evaluation of the ECT treatment.

**Disclosure of Interest:**

None Declared

